# Clinical Application of CT-Guided Percutaneous Microwave Ablation for the Treatment of Lung Metastasis from Colorectal Cancer

**DOI:** 10.1155/2017/9621585

**Published:** 2017-10-31

**Authors:** Lin Li, Ketong Wu, Haiyang Lai, Bo Zhang

**Affiliations:** Department of Radiology, The Sixth Affiliated Hospital of Sun Yat-sen University, Guangzhou 510655, China

## Abstract

**Objective:**

The aim of our research is to explore the clinical efficacy and safety of CT-guided percutaneous microwave ablation (MWA) for the treatment of lung metastasis from colorectal cancer.

**Materials and Methods:**

CT-guided percutaneous MWA was performed in 22 patients (male 14, female 8, mean age: 56.05 ± 12.32 years) with a total of 36 lung metastatic lesions from colorectal cancer between February 2014 and May 2017. Clinical data were retrospectively analyzed with respect to the efficacy, safety, and outcome.

**Results:**

Of the 36 lesions, 34 lesions (94.4%) reduced obviously with small cavitations or fibrous stripes formed and had no evidence of recurrence during follow-up. The volume of the other 2 lesions demonstrated local progression after 6 months by follow-up CT. The primary complications included pneumothorax (28%), chest pain (21%), and fever (5%). These symptoms and signs were obviously relieved or disappeared after several-day conservative treatment. The mean follow-up of the patients was 25.54 ± 12.58 months (range 2–41 months). The estimated progression-free survival rate was 94.4%.

**Conclusion:**

Our results demonstrate that CT-guided percutaneous MWA appears to be an effective, reliable, and minimally invasive method for the treatment of lung metastasis from colorectal cancer. This trial is registered with ChiCTR-ORC-17012904.

## 1. Introduction

Colorectal cancer (CRC) is a common malignancy, with approximately 1,360,000 new cases each year worldwide [1, 2]. It is the third most commonly diagnosed cancer in males and the second in females [1]. With increasing incidence and mortality, it has been an important cause of death and is a major public health problem [3]. Colorectal cancer can get good treatment and survival prognosis by surgical resection [4, 5], but distant metastasis can occur in more than 50% of patients which is the main reason leading to poor outcomes [6, 7].

Lung metastases can develop in 5 to 15% of those CRC patients [6–9]. Patients with untreated metastatic disease have a median survival of less than 10 months and a 5-year survival rate of less than 5% [8]. Since Gonzalez et al. [10] first reported the pulmonary resection for colorectal metastases, surgical resection seems to have become the main radical treatment available for pulmonary metastases with low morbidity and mortality. However, only a minority of patients will be cured [7]. We need to carry out targeted treatment because of the large trauma, wide range of the surgical resection, complicated postoperative care, and the patient's poor acceptance or tolerance for surgical treatment.

Minimally invasive interventional treatment, especially thermal ablation, has been increasing in popularity for lung metastases; it not only acts as a primary treatment but also as adjunctive therapy to external radiation [11]. Thermal ablation mainly includes radiofrequency ablation (RFA) and microwave ablation (MWA), both of them are high-temperature-based modalities, which lead to coagulation necrosis in tumor tissues [12–14]. Currently RFA is a relatively common procedure in tumor area; however, several clinical studies have suggested that MWA is also effective with many advantages [12]. MWA can generate higher temperatures in larger zones than RFA which has been proved in animal testing [15]. Like RFA, MWA allows for flexible approaches to treatment, including percutaneous, laparoscopic, and open surgical access [14]. The purpose of this study was to explore the clinical efficacy and safety of computed tomography- (CT-) guided percutaneous MWA for the treatment of lung metastasis from colorectal cancer.

## 2. Materials and Methods

### 2.1. Clinical Material

From February 2014 to May 2017, twenty-two patients (14 males, 8 females) with 36 pulmonary metastases from colorectal cancer were treated by CT-guided percutaneous MWA. The study protocol was approved by the Institutional Ethics Review Board of our hospital. Written informed consent prior to percutaneous MWA was obtained from each patient.

All patients underwent complete resection of primary colorectal cancer (R0), and the primary tumor was confirmed as adenocarcinoma by pathology. During follow-up, all of the pulmonary lesions in our study showed a malignant tendency; according to the American College of Chest Physicians (ACCP) in 2013 and Asian consensus guidelines on the diagnosis of lung nodules [16, 17], a consensus clinical diagnosis of pulmonary metastases from colorectal cancer was made by a multidisciplinary team including radiologists, thoracic surgeons, medical oncologists, and colorectal surgeons. One of the cases was confirmed as pulmonary metastases by fibrotic bronchoscope biopsy.

### 2.2. Indications or Contraindications

The guidelines for the diagnosis and treatment of pulmonary metastases are absent at present, so we can draw on the inclusion and exclusion criteria for thermal ablation therapy for lung metastases from other reports [18–24]. All patients were assessed by a multidisciplinary team including interventional radiologists, thoracic surgeons, medical oncologists, and colorectal surgeons. They were selected according to the following criteria: patients with poor physical fitness, medical reasons of poor candidates for surgery (e.g., limited cardiopulmonary reserve), number of lesions per patient less than five, the maximum diameter less than 3.5 cm, the distance between lung metastases and cardiac large blood vessels more than 0.5 cm, and a life expectancy of more than 6 months.

The exclusion criteria included uncontrolled primary malignancy, extrapulmonary metastasis, the presence of more than five lesions, lesions with a maximal axial diameter greater than 3.5 cm, tumor infiltration of the chest wall or mediastinal structures, uncontrolled coagulopathy (platelet count < 75,000/mL; international normalized ratio ≥ 1.5), confirmed bacteremia, serious failure of the function of important organs (heart, liver, lung or kidney), severe anemia, and metabolic disturbance unable to be corrected in the short term.

### 2.3. Preparation before Ablation

This preprocedural evaluation is very similar to a surgical evaluation. In general, most patients who can tolerate CT-guided needle biopsy are good candidates for pulmonary MWA [14]. To reduce potential complications of sedation-induced nausea and aspiration of gastric contents, all patients were treated after an overnight fast. For some basic diseases, such as hypertension and heart disease, we made patients take antihypertensive and cardiac medications in the morning. An abridged physical examination would be performed before operation, including basic biochemical tests, vital sign assessment, and cardiopulmonary function assessment. Venous catheter before surgery would be placed as well. It was worth mentioning that we did not routinely use prophylactic antibiotics and general anesthesia during operation. Percutaneous MWA was performed with the conscious sedation (morphine or flurbiprofen acetate injection). Occasionally, general anesthesia was used in patients who may not tolerate the MWA heating or pain with conscious sedation alone [14].

### 2.4. Operation Process

All ablation process of lung metastases was performed under the guidance of a spiral CT (Optima CT660, General Electric Company, America). The position of the patient in the examination bed was determined by the location of the lesion in the lung (supine or prone position), which was all localized with preprocedural CT scan. First, we began the preprocedural CT scan with 5 mm contiguous slices and defined the exact skin entry site from the CT images located, then marked it on the skin. In order to keep the patient awake and alleviate the pain during surgery, 2% lidocaine hydrochloride was used both intradermally and deeper to the pleural surface for local anesthesia.

Percutaneous entry route was chosen on the basis of tumor size, morphology, location, adjacent structures, and access route [25]. In addition, we chose one or more punctures according to the size of the tumor; in general, lesions greater than 3.5 cm require multiple ablation probes to make a more thorough necrosis. For MWA, we used a commercially available system (KY-2450B, Microwave Energy Nanjing Kangyou Applied Research Institute, China) and a 14-gauge cooled shaft antenna. Microwave antenna slowly penetrated the lesion in accordance with the direction and angle of the proposed preoperative. To ensure tumor adequate necrosis, the microwave antenna is positioned parallel to the long axis of the lesion as far as possible and position the ablation antenna beyond the edge of the lesion 0.5–1.0 cm, then confirm the above mentioned by CT scans. Internal cooling of the antenna shaft was performed with a peristaltic pump that recirculated room-temperature normal saline at a rate of 50–60 mL/min to prevent thermal injury along the proximal antenna shaft. The power of microwave generator was generally set at approximately 40–60 W. The ablation time for 2.0-3.5 cm tumors was 4–8 minutes.

During the ablation procedure, the patient's vital signs (respiration, heart rate, pulse, blood pressure, and oxygen saturation) were closely monitored. At the end of ablation, CT scan was performed again to evaluate the immediate necrotic conditions of the tumor and the surrounding lung tissue and to examine whether there were any complications, such as pneumothorax or hemorrhage. A moderate or large pneumothorax (1 cm of separation between visceral and parietal pleura) found at postprocedural imaging was initially treated by aspiration with a 7-F central venous catheter. If the pneumothorax remained unresolved, an 8.5-F pigtail drainage system or underwater-sealed bottle was applied.

### 2.5. Follow-Up Method and Statistical Analyses

Imaging immediately after ablation can be used to rule out typical complications (pneumothorax, hemorrhage) and to document the effects of treatment, and we performed chest X-rays at 4 hours and 24 hours to rule out delayed onset pneumothorax [12]. In this study, CT scans were performed every 2 to 3 months as the reference value of efficacy evaluation until the end of follow-up, if necessary, PET-CT as well. A modified Response Evaluation Criteria in Solid Tumors (RECIST) criterion incorporating CT scan was quoted to evaluate initial response to our treatment [26].

Local tumor progression was mainly indicated by variation of the enhancement pattern and size of the tumor. That is, we find that when compared with the previous imaging examination, lesions tend to gradually become larger and accompanied by irregular edges. In addition, the ablation lesions may show uneven, scattered, nodular, or eccentric enhancement in the contrast enhancement. Conversely, the tumor was considered to be completely treated if the entire ablation lesion was not significantly enhanced and dwindle in size [26–28]. Additionally, the estimated progression-free survival rate was evaluated using the Kaplan-Meier analysis.

## 3. Results

### 3.1. Local Tumor Response and Survival

**Figure 1:**
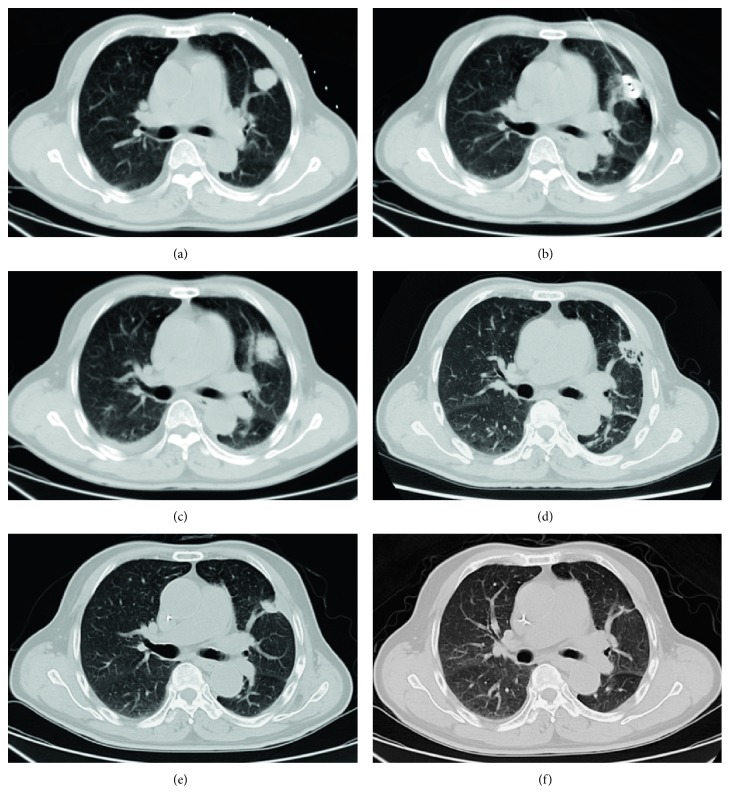
A 73-year-old man with lung metastasis from colorectal carcinoma in left upper lobe with an axial diameter of 2.3 cm (a). Ablation was performed after accurate insertion of MWA probe into the focus of tumor (b). The immediate postoperative CT scan showed that the lesion enlarged slightly with ill-defined and irregular margin (c). The lesion was shrunk gradually and replaced by fibrous tissue during 1 (d), 3 (e), and 6 (f) months follow-up.

**Figure 2:**
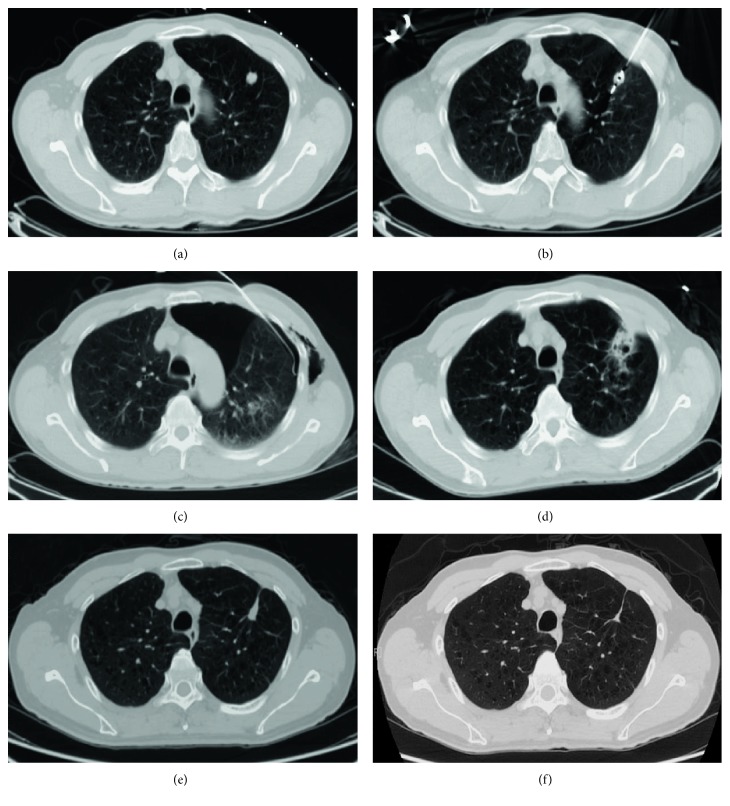
A 62-year-old man with lung metastases from colorectal carcinoma in the left upper lobe with a diameter of 1.5 cm (a). Ablation was performed after accurate insertion of MWA probe into the focus of tumor (b). The immediate postoperative CT scan showed pneumothorax (lung compressed approximately 40%), and thoracic cavity closed drainage was conducted immediately (c). The lesion enlarged slightly with ill-defined margin and air-filled cavity on one month follow-up (d). During 3 (e) and 6 (f) months follow-up, the lesion was shrunk gradually and replaced by fibrous tissue with no evidence of tumor remnants.

**Figure 3:**
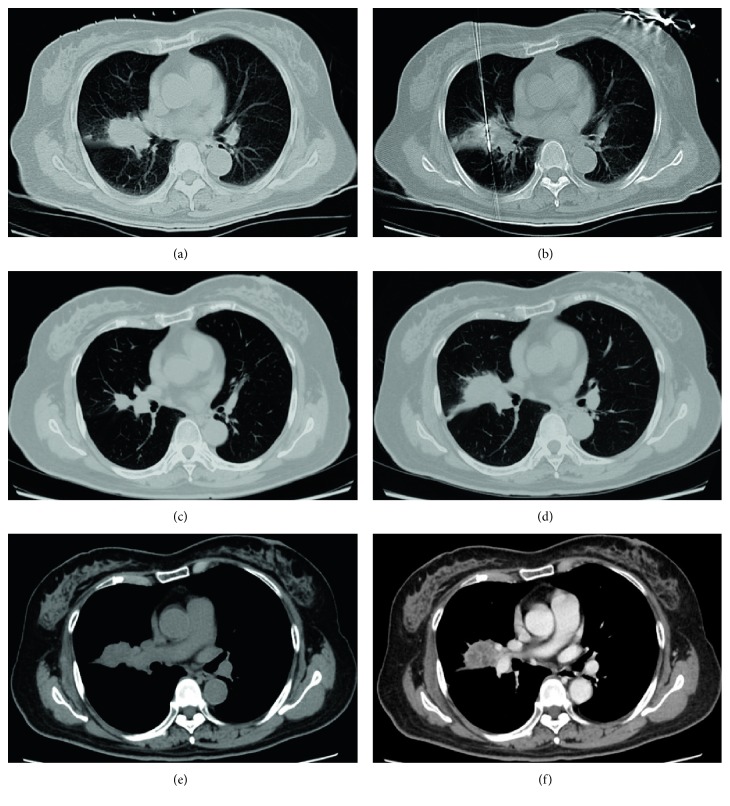
A 66-year-old female with nodular lung metastatic lesion with a diameter of 2.5 cm (a). Ablation was performed after accurate insertion of MWA probe into the focus of tumor (b). The lesion reduced obviously with some fiber cable around it on 3 months follow-up (c). However, the volume of the tumor increased again on 6 months follow-up ((d) lung window; (e) soft tissue window; (f) contrast-enhanced CT), which was consistent with the description of local tumor progression.

**Figure 4:**
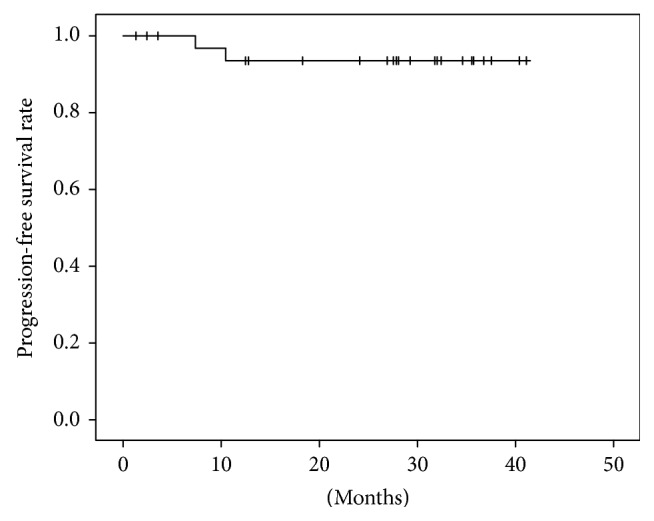
Kaplan-Meier curves for progression-free survival rate in 22 patients with 36 pulmonary metastases from colorectal cancer after MWA treatment.

The clinical features of our patients were shown in Table 1. Lesions were found in a short time after ablation by CT follow-up (especially within 24–48 hours) and showed honeycomb-like or empty-like changes, which ground-glass opacity around the lesion called “cap badge signs” can be seen [12, 14] (Figure 1). After 1 month, the ground-glass opacity around the lesion was basically absorbed, the lesion was enlarged compared with preoperative; besides, cavities can be found in some lesions (2 lesions occurred in our study), and significant enhancement in CT scan was hardly observed. Within 3–6 months after operation, the lesions were gradually reduced and no enhancement on CT contrast-enhanced scan, and some of the lesions were surrounded by fibrous bundles (Figure 2). Six months after the operation, the volume of two lesions increased in varying degrees, and the edge of them was irregular, which showed markedly inhomogeneous enhancement (CT value increased both more than 15 Hu) on CT scans [26], suggesting local progression of tumor (Figure 3). The mean follow-up of the patients was 25.54 ± 12.58 months (range 2–41 months). The estimated progression-free survival rate was 94.4% (Figure 4).

### 3.2. Complications

In our study, 22 patients (mean age: 56.05 ± 12.32 years, range 25–73 years) with 36 lesions (1.6 lesions per patient) were successfully treated with 39 times of microwave ablation under the guidance of CT. One patient with good pulmonary reserve was treated with ipsilateral pulmonary lobe simultaneous ablation of both lesions. Treatment-related complications were defined as related symptoms that occurred within 30 days after ablation, and severity was defined in accordance with the Common Terminology Criteria for Adverse Events (CTCAE) version 4.0 [24, 29]. We believed that the grade 1-2 did not require invasive disposal, but grade 3-4 must be treated immediately, that is, closed-chest drainage. The complications in 39 times ablation were demonstrated in Table 2. Pneumothorax occurred eleven times (28%) after ablation, of which five times (13%) the amount of pneumothorax exceeded over 40% and received thoracic cavity closed drainage (Figure 2). The other six times (15%) with mild pneumothorax had no requirement for treatment. Eight times (21%) after ablation, the patients complained chest pain; we considered it as a reaction to postoperative which disappeared after given pain medication and other symptomatic treatment. Fever occurred two times (5%) after ablation, and the temperature is both below 38.8°C. It may be related to the absorption of coagulative necrosis of the tumor or local inflammatory reaction; the fever and other symptoms returned to normal after conservative treatment. No pleural effusion, hemothorax, and hemoptysis occurred. No needle track implantation and no patient deaths occurred within 30 days after the operation.

## 4. Discussion

Surgical resection is the first-line treatment for lung metastases from colorectal cancer [12]. Many patients cannot be candidates for surgical treatment because of the great injury, complicated postoperative care, and the patient's poor acceptance or tolerance, which provided a basis to carry out targeted treatment. With the development of minimally invasive technique, thermal ablation has been gradually applied to the treatment of solid tumors [14]. RFA and MWA are the most common types of thermal ablation. Although RFA is a procedure which has much clinical experience to date, several clinical studies have suggested that tumor heating with microwave energy is also effective [11–14]. In contrast to RFA relying on electric currents and conduction through tissue, the benefits of MWA are higher constant intratumoral temperatures, faster ablation times, and the possibility of using multiple antennas to treat multiple lesions simultaneously, which make MWA more suitable for tissues with higher impedance and high water content, especially for pulmonary metastases [11, 13].

In this study, most of the lesions were gradually reduced and no enhancement was found in enhanced CT scans, and fibrous bundles could be seen around the lesion or local scarring formed in the site of the lesions, implying that the tumor might have been completely destroyed. However, the volume of two ablation lesions increased with irregular margin and marked inhomogeneous enhancement on CT scans after 6 months, which suggested local progression of tumor [24, 27, 30].

Based on the small number of our series, MWA could significantly destroy lung metastasis from colorectal cancer. In order to explore the value of MWA more comprehensively, a brief review of the published literature summarizing MWA in the treatment of lung metastasis from colorectal cancer was performed. By searching the terms “lung/pulmonary metastases,” “colorectal cancer,” and “microwave ablation,” the related literatures within the recent ten years via the PubMed database were analyzed. To identify additional articles and case reports, the secondary references of these publications were also reviewed. As of the time of manuscript preparation, a total of 6 articles published in English involving MWA in the treatment of lung metastasis from colorectal cancer were available from PubMed. As shown in Table 3 [21, 24, 25, 30–32], a total of 214 lesions in 125 patients were identified and analyzed based on their clinical characteristics and outcome, demonstrating the benefit of MWA in these patients. On the whole, MWA may be an effective means of treating lung metastasis from colorectal cancer.

During the process of ablation, chest pain was one of the most common symptoms that could be greatly relieved through intramuscular injection of 10 mg morphine. The pain may be strongly associated with the lesion near to pleura which was stimulated by needle puncturing and heat from ablation. In addition, the severe decrease of heart rate and blood pressure caused by vasodepressor reflex was another question which needs immediate attention [33]. Generally, the change of heart rate and blood pressure can be gradually restored by intramuscular injection atropine and stop ablation.

As previously reported [21, 24, 25, 30–32, 34], pneumothorax was the most common complication after microwave ablation for lung metastases. There were total 11 cases of pneumothorax after ablation, but only 5 cases needed closed thoracic drainage for 2 days. The mild pneumothorax in the other 6 cases was completely absorbed by itself. In our study, no delayed pneumothorax was found, and no complications such as pleural effusion, hemorrhage, and other serious complications such as ARDS and pulmonary infarction were observed. However, serious hemoptysis should be constantly paid attention. It is very important to select appropriate patients and to avoid large vessels and bronchus during puncture to reduce the risk of hemoptysis.

There are several limitations in this study. First, the data were analyzed retrospectively and as such the study is subject to the inherent limitations of retrospective studies. Second, because of the rigorous inclusion and exclusion criteria, this single-center, observational, preliminary study suffered from a small sample size and short-term follow-up. Third, we do not report the absolute value of the cost-effectiveness and complications compared to conventional surgical treatment. And, this article was not compared with other competing treatment modalities, the result may be biased.

In summary, based on the present results and a brief review of the literature, there is some agreement that CT-guided percutaneous MWA appears to be an effective, reliable, and minimally invasive method for the treatment of lung metastasis from colorectal cancer. Further prospective randomized studies are required to demonstrate the cost-effectiveness and safety compared to conventional surgical resection.

## Figures and Tables

**Table 1:** Clinical features of the patients.

Clinical parameters	Number of patients, *N* (%)
*Sex*	
Male	14 (57.9)
Female	8 (42.1)
*Age* (*years*)	
Range	25–73
Mean	56.05 ± 12.32
*Unilateral/bilateral*	
Unilateral	18 (81.8)
Bilateral	4 (18.2)
*Lesion size* (*cm*)	
≤3.5	22
>3.5	0
*Number of lesions*	
1	14 (63.6)
>1	8 (36.4)
*Primary tumor*	
Rectum	15 (68.2)
Colon	7 (31.8)
*CEA*	
Negative (≤5 ng/mL)	9 (40.9)
Positive (>5 ng/mL)	13 (59.1)
*Total*	**22**

**Table 2:** Complications of 39 times ablation.

Complication^∗^	Rate(%)^a^
Pneumothorax	11 (28%)
Mild^b^	6 (15%)
Severe^c^	5 (13%)
Chest pain	8 (21%)
Fever	2 (5%)
Hemothorax	0
Hemoptysis	0
Pleural effusion	0

^∗^Classification consisted of the Common Terminology Criteria for Adverse Events version 4.0 [29]. ^a^The rate was calculated by dividing the times of symptom in parentheses by the total times of ablation (*n* = 39). ^b^Grades 1-2, no chest tube required. ^c^Grades 3-4, chest tube required.

**Table 3:** Literatures related to the ablation of lung metastases from colorectal cancer in recent years.

Author institution	Recruitment period	Characteristics of patients	Patient with LM from CRC	Key outcome^n^
Wolf et al. 2008 [24]	2003–2006	*N* = 50; 28 men, 22, women; mean age 70 years	9	Residual disease and recurrent disease of the ablation was 22% and 26%, respectively.
Vogl et al. 2001 [30]	2007–2010	*N* = 80; 30 men, 50 women; mean age 59.7 years	40 (58 lesions)	New metastases developed in patients with colorectal cancer 7.5%.
Lu et al. 2012 [25]	2005–2008	*N* = 69; 45 men, 24 women; mean age 65 years	7	Local progress of masses appeared 21.74%.
Wolf et al. 2012 [31]	2009-2010	*N* = 10; 6 men, 4 women; mean age 71 years	1	The risk of local disease progression was decreased.
Raspanti et al. 2014 [32]	2009–2012	*N* = 21, 36 nodules	21 (36 lesions)	Residual disease was observed about 2.8%.
Vogl et al. 2016 [21]	2000–2014	*N* = 109; 71 men, 38 women; mean age 68.6 years	47 (103 lesions)	Local progress of masses appeared 12%.

LM: lung metastases; CRC: colorectal cancer. ^n^The histopathologic type of the metastasis was not a statistically significant factor when correlating with the ablation result [25, 30].
